# The effect of *Acacia nilotica* bark extract on growth performance, carcass characteristics, immune response, and intestinal morphology in broilers as an alternative to antibiotic growth promoter

**DOI:** 10.5713/ab.22.0284

**Published:** 2023-01-11

**Authors:** Muhammad Umer Zahid, Anjum Khalique, Shafqat Nawaz Qaisrani, Muhammad Ashraf, Ali Ahmad Sheikh, Muhammad Umar Yaqoob

**Affiliations:** 1 Department of Animal Nutrition, University of Veterinary and Animal Science, Lahore 54000, Pakistan; 2 Department of Pharmacology and Toxicology, University of Veterinary and Animal Sciences, Lahore 54000, Pakistan; 3 Institute of Microbiology, University of Veterinary and Animal Sciences Lahore 54000, Pakistan; 4 College of Animal Science, Zhejiang University, Hangzhou 310058, China; 5 Provincial Key Agricultural Enterprise Research Institute of King Techina, Hangzhou King Techina Feed Co., Ltd., Hangzhou 311107, China

**Keywords:** *Acacia nilotica*, Antioxidant Capacity, Bark Extract, Blood Biochemistry, Growth Performance, Intestinal Morphology

## Abstract

**Objective:**

Present study was designed to evaluate the efficacy of *Acacia nilotica* bark extract as an alternative to antibiotic growth promoters in broilers.

**Methods:**

Six hundred, day-old broiler chicks were randomly divided into six groups (NC, without any supplementation; AB, NC+Zinc Bacitracin; PB, NC+Safmannan; ANBE1, NC+*A. nilotica* bark extract 0.1%; ANBE3, NC+*A. nilotica* bark extract 0.3%; ANBE5, NC+*A. nilotica* bark extract 0.5%), with ten replicates per group (10 chicks/replicate) and feeding trial was lasted for 35 days.

**Results:**

Results showed that weight gain (1,296.63 g) and feed conversion ratio (FCR, 1.59) of AB was better than NC, during the finisher phase. Overall FCR of AB (1.53), PB (1.54), and ANBE5 (1.54) was significantly (p<0.05) better than NC. From carcass parameters relative weight of wing and heart were highest in ANBE3 (2.5% and 1.51%, respectively). Significantly (p<0.05) highest blood glucose level was observed in NC (264.5 mg/dL) and highest albumin concentration was found in AB (1.46 mg/dL). In addition, antibody titer levels against ND and IBD were higher in ANBE5 than NC, while higher relative weight of bursa was observed in ANBE3 than NC. The villus height to crypt depth ratio in all experimental groups was better than NC.

**Conclusion:**

*Acacia nilotica* bark extract could be a suitable alternative to antibiotic growth promoters to support the growth in broilers.

## INTRODUCTION

Antibiotics have been used for decades for treatment of bacterial infections and to improve production efficiency in the poultry industry. Notwithstanding the benefits of antibiotics, developing bacterial resistance against frequently used antibiotics is a global concern. Improper use of antibiotics for long duration have led to antibacterial resistance and development of multidrug-resistant strains of bacteria [1], which could be transferred to other farm animals and humans [2]. Use of food contaminated with antibiotic resistant bacteria is a key point to consider because it might become challenging and costly to treat infection caused by such types of bacteria [3,4]. Several European countries have banned the use of antibiotics at sub-therapeutic levels [5]. In those countries there is a decrease in the growth performance of poultry birds and increase in rate of mortality due to bacterial infections such as salmonellosis, necrotic enteritis and colibacillosis [6]. Based on these premises, farmers, nutritionists, and pharmacists have been searching for suitable alternative which have no residual effect and maintain the growth performance of poultry birds at optimum levels [7].

Natural plant and herbal products could be novel antimi crobial agents with potentially new modes of action [8]. Active principles found in plants could be a safe and effective alternative to synthetically produced antimicrobial agents. The active principles found in different plants are volatile essential oils, alkaloids, phenols, resins, phenolic oleosins, glycosides, tannins, steroids, and terpenes which have antibacterial activities against different bacteria [9] and have antioxidant effect [10]. Among medicinally important plants *Acacia nilotica* has its own importance due to its many medicinal uses and antioxidant activity. Work has been done on the extract of different parts of *A. nilotica* which showed that it contains many phytogenic feed additives which have beneficial effects not only in poultry but also in humans [11]. *A. nilotica* has been reported to improve the growth performance in poultry [12]. *A. nilotica* could improve the health status of poultry birds and increase the bioactive compounds index in poultry meat [13]. It has been reported that extract of different medicinal plants improves the nutrients digestibility by enhancing the secretions of digestive enzymes which led to improved growth performance in poultry birds [14]. They also improved gut health by enhancing the permeability of cell membranes for nutrient absorption and killing the pathogenic bacteria [14,15]. Keeping in view the growing concern of antibiotic resistance and importance of *A. nilotica* in poultry, present study was planned to evaluate the efficacy of the use of bark extract of *A. nilotica* on different biological parameters in broilers and to compare it with antibiotics.

## MATERIALS AND METHODS

### Institutional review board statement

The protocol of this experiment was approved by committee of Ethical Handling of Experimental Birds, University of Veterinary and Animal Sciences, Lahore, Pakistan, for the care and use of experimental birds with approval number: DAS/358, Dated 03/05/2021.

### Bark collection and analysis

Stem bark of mature and healthly *A. nilotica* was collected from Lahore and Kasur, Punjab, Pakistan and washed thoroughly under running water to remove dirt, followed by rinsing with sterilized distilled water. Bark was oven dried at low temperature and ground with an efficient grinding machine with a sieve of 2 mm. Ground bark powder (100 g) was extracted with absolute ether (600 mL) through Soxhlet Extraction technique [16]. Maximum extract was collected after 10 to 12 extraction cycles. Extract was concentrated by using rotary evaporator under high pressure (>60 Pascal) and controlled temperature (40°C to 50°C). The extract was then placed in oven at 40°C for four days for maximum removal of moisture. The extract then placed in plastic zip bags at −20°C till further analysis or use. Phytochemical analysis of extract was done for presence or absence of tannins, alkaloids, terpenoids, glycosides, saponins, steroids and flavonoids, following previous methods [16–18]. Bark extraction and phytochemical analysis were performed in the Department of Animal Nutrition, University of Veterinary and Animal Sciences, Lahore, Pakistan.

### Animals and study design

Six hundred, day-old broiler chicks (Cobb-500) were divided into six groups (NC, AB, PB, ANBE1, ANBE3, and ANBE5) consisted of ten replicates each (10 chicks per replicate). NC, negative control with no supplementation; AB, positive control, NC+Zinc Bacitracin; PB, probiotic supplemented group, NC+Safmannan; ANBE1, NC+*A. nilotica* bark extract 0.1%; ANBE3, NC+*A. nilotica* bark extract 0.3%; ANBE5, NC+*A. nilotica* bark extract 0.5%. *A. nilotica* bark extract was sprayed after pelleting on to the cooled pellets. Feeding trial was conducted at the R&D farm of Sultan Feed Pvt. Ltd., Sargodha, Pakistan, for 35 days, divided into two phases: starter phase 1 to 21 days; finisher phase 22 to 35 days. Diet of the negative control group was formulated according to “Cobb-500 Nutritional Requirement Manual Guide, 2018” (Table 1). Free access to feed and water was provided to the birds throughout the experiment.

### Samples collection and measurements

#### Growth performance

During feeding trial feed intake and weight gain were recorded on weekly bases. Data were arranged to calculate average feed intake, weight gain and feed conversion ratio (FCR), during starter and finisher phases and on overall basis.

#### Carcass characteristics

At the end of the experiment, 1 bird/replicate was selected and slaughtered to determine the effect of dietary treatments on carcass characteristics. Live body weight of selected birds was recorded and carcass weight was also recorded after slaughtering. Dressing percentage was calculated from data of live body weight and carcass weight. While relative weight of different parts (breast, thigh, and wing) and internal organs (heart, liver, and gizzard) were calculated as a percent of carcass weight.

#### Blood chemistry

Blood samples (about 3 mL form each bird) were collected from jugular vein at the time of slaughtering of birds and serum was separated by centrifugation, which was collected in Eppendorf tubes and stored at −15°C to −20°C until further analysis. Serum was analyzed for total protein, albumin, globulin, blood glucose and triglyceride levels using respective kits [19].

#### Immune response

Immune response was analyzed by determining the levels of haemagglutination inhibition titer against Newcastle disease virus (NDV) and ELISA titer level against infectious bursal disease virus (IBDV) [20]. In addition, immune organ indexes were recorded.

#### Jejunum morphology

Jejunum was separated from slaughtered birds and about 2 cm part was cut and rinsed with normal saline and stored in 10% neutral formalin solution. Preserved samples was used to prepare slides by sectioning it using a microtome (5 μm). Hematoxylin and eosin staining was done followed by morphometric analysis using a microscope having a computer assisted morphometric system (Nikon Corporation, Tokyo, Japan).

### Statistical analysis

Data collected for all parameters were arranged and expressed as mean±standard error of the mean. All data were analyzed through one-way analysis of variance using SPSS 26. Tukey’s test was used to determine statistical difference among the treatments at significance level of p<0.05.

## RESULTS

Screening of bark extract of *A. nilotica* revealed that it contains tannins, alkaloids, terpenoids, glycosides and saponins. On the other hand, steroids and flavonoids were not detected in the extract (Table 2).

### Growth performance

No significant effect of dietary treatments was observed on all parameters of growth performance during starter phase. During finisher phase weight gain and FCR in AB was significantly (p<0.05) better than NC but similar to *A. nilotica* bark extract supplemented groups. Similar effect was observed on weight gain during overall experiment. However, FCR of AB, PB, and ANBE5 was significantly better than NC on overall bases (Table 3).

### Carcass characteristics

Dressing percentage varied from 68.29% to 69.43% with highest in PB and lowest in NC. Numerically highest relative breast weight was observed in AB (23.50%) followed by PB (23.26%) and lowest in ANBE5 (22.22%). Highest (p<0.05) relative weight of wing (2.5%) was observed in ANBE3 which was statistically similar to other groups, except ANBE5. Higher (p<0.05) relative weight of heart (1.51%) was found in ANBE3 than NC, AB, and PB groups (Table 4).

### Blood biochemistry

Effect of dietary treatments was also evaluated on different parameters of blood biochemistry and the result exhibited no significant difference among the groups except glucose and albumin levels (Table 5). Significantly (p<0.05) highest blood glucose level was observed in NC (264.5 mg/dL) and lowest in ANBE5 (213.75 mg/dL). Total protein levels were not affected; however, highest albumin levels were found in AB (1.46 mg/dL) followed by NC (1.36 mg/dL) and lowest was observed in ANBE5 (1.20 mg/dL). Levels of triglyceride in blood serum varied from 48.57 to 61.25 mg/dL in ANBE1 and NC, respectively.

### Immune response

**Figure 1:**
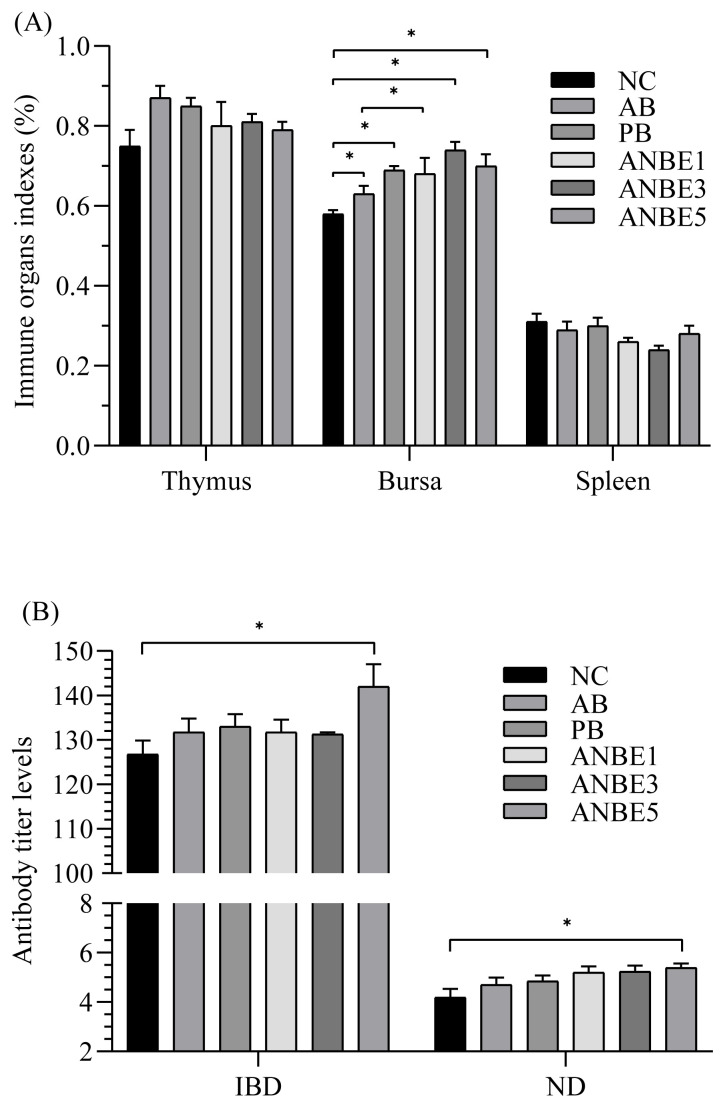
Effect of *Acacia nilotica* bark extract on immune response in broiler. (A) Relative weight of immune organs; (B) antibody titer levels. NC, negative control; AB, antibiotic supplemented group; PB, probiotic supplemented group; ANBE1, ANBE3, and ANBE5 were supplemented with *A. nilotica* bark extract at the levels of 0.1%, 0.3%, and 0.5%, respectively. Statistic difference from corresponding control value for a given parameter is annotated by: * p<0.05.

Immune response was analyzed by evaluating the effect of dietary treatments on relative weight of immune organs (Figure 1A) and antibody titer levels against NDV and IBDV (Figure 1B). Significantly (p<0.05) higher antibody titer levels against NDV and IBDV were observed in ANBE5 (5.38; 142) than NC (4.18; 126.75), however it was statistically similar to other groups. Relative weight of bursa in ANBE3 (0.74%) was statistically similar to other experimental groups (0.68% to 0.70%) and PB (0.69%) but significantly (p<0.05) higher than NC (0.58%). Relative weight of spleen and thymus was statistically similar among the different groups.

### Intestinal morphology

No significant effect of dietary treatments was found on villus height but crypt depth and villus height to crypt depth ratio were affected (Table 6). Villus height varied from 1,201.60 μm to 1,305.72 μm, with lowest in NC and highest in ANBE5. Highest crypt depth was found in NC (234.83 μm) and lowest in ANBE5 (204.93 μm). The villus height to crypt depth ratio in all experimental groups (5.95 to 6.37) and AB (6.34) groups was similar but statistically (p<0.05) higher than NC (5.13).

## DISCUSSION

Extract of different parts of *A. nilotica* have been used in traditional medicines in different countries including Chinese, Unani, Ayurvedic and Egyptian for centuries because of the presence of different bioactive secondary compounds. Extracts of different parts of *A. nilotica* have bioactive compounds which support the health status in animals and humans and used for prevention and treatment of different diseases. Tannins, saponins, and flavonoids are used in digestive disorders; saponins and triterpenoids in cancer and polyphenols as antioxidants [21]. Screening analysis of bark extract confirmed the presence of tannins, alkaloids, terpenoids, glycosides and saponins, while steroids and flavonoids were absent. Results showed that overall weight gain and FCR were improved with *A. nilotica* bark extract supplementation in broilers, while carcass characteristics were not affected. Results of growth performance suggested that boilers could grow better in the absence of antibiotics with supplementation of *A. nilotica* bark extract and these results are in line with Abudabos et al [22], who stated that *A. nilotica* extract could effectively support the growth performance in broilers similar to antibiotics. Present results agree with that of Festus et al [23], who stated that herbal extract of *A. nilotica*, *Moringa oleifera* and wild mushroom could be used as a replacement for AGPs without affecting growth performance and carcass parameters in broilers. Similarly, Ngambi et al [12] stated that supplementation of leaf extract of *A. nilotica* had no significant effect on feed intake in broilers however fat weight was reduced. Present results are in line with previous study of Marimuthu and D’Souza [24], who stated that commercial choline chloride could be replaced with herbal mixture of *Curcuma longa* and *A. nilotica* in broilers. Herbal mixture supported the growth-related parameters as done by choline chloride. In another study, inclusion of 10% *A. nilotica* seed meal significantly improved weight gain and FCR with lowest feed cost per unit weight gain in broilers [25]. *A. nilotica* leaf meal supplementation also increase the weight gain and economic efficiency with suitable FCR in broilers [26]. Improved growth performance and FCR during this experiment and previous studies [13–15] might be due to presence of bioactive compounds in extract which stimulate the production of digestive enzymes [15] and improved the nutrients’ digestibility [27,28].

Analysis of blood biochemistry showed that *A. nilotica* extract reduced the blood glucose levels and enhanced albumin levels in broilers, while other components remained unaffected. Similarly, albumin levels increased in boilers with supplementation of phytogenic additives in broilers [15]. Previous study confirmed that *A. nilotica* extract controlled blood sugar level in hyperglycemic rats and maintained normal blood lipid profile [29]. Another study stated that intraperitoneal injection of *A. nilotica* bark extract is an effective way to control blood sugar level in diabetes model induced in mice [30]. Additional study conducted on rabbits, in which diabetes was induced by alloxan, suggested that *A. nilotica* pods extract significantly reduced the blood glucose level and maintained the blood lipids profile [31]. Olorede et al [25] stated that use of *A. nilotica* seed meal at the levels of 20% significantly increased hemoglobin, packed cell volume and red blood cells without compromising the body weight in broilers.

Immune response was analyzed by evaluating the effect of dietary treatments on immune organs indexes and antibody titer levels against ND and IBD. Results suggested that *A. nilotica* extract has some role in stimulation of immune system, as antibody titer levels were enhanced by *A. nilotica* extract supplementation and relative weight of bursa was also improved and results are supported by previous results that *A. nilotica* pod extract stimulated the immune system in rats [32]. *A. nilotica* pod extract supplementation enhanced the counts of white blood cells [32], which are the primary line of defense against infection and tissue damages [33], which suggested that bioactive compounds of extract stimulate the immune system to protect the body of the host animal. Another study stated that *A. nilotica* extract boosted the proliferation of splenocytes [34] and these findings were also supported by *in vitro* analysis [35]. Enhanced proliferation of splenocytes is the indication of activation of cellular immune response in animals [34] which are suppressed by immune suppression activities of pathogenic microorganisms [36].

Gut health has an important role maintaining the health status as it is the main site of digestion and absorption of nutrients. Intestinal morphological parameters (villus height, crypt depth, and their ratio) are indicators of gut health, its maturity and functionality [37] which are mainly affected by dietary manipulation [38]. Improved morphological parameters suggested better gut health required to improve the feed efficiency and support the health of animals. In present study intestinal morphology was improved by *A. nilotica* bark extract supplementation, which might be the reason for improved FCR and better weight gain in broilers. Similar to AGPs, *A. nilotica* extract could induce modification in immune system and microbial load at intestinal levels. Decrease in microbial load and reduction in unnecessary use of energy lower the energy wastage and enhance growth performance. In addition, strong immune system with reduced load of pathogenic microbes reduces the incidence of diseases in broilers [39].

## CONCLUSION

Based upon present results it is concluded that *Acacia nilotica* bark extract could be a suitable alternative to antibiotic growth promoters to support the growth in broilers. *Acacia nilotica* bark extract enhances the immunity and supports the gut health in boilers.

## Figures and Tables

**Table 1:** Composition of negative control diets

Items	Starter diet	Grower diet
Ingredients composition (%)		
Maize	55.61	59.13
Soybean meal	25	20
Guar meal	3	3
Canola meal	10	10
Corn gluten 60%	0.11	1.89
Veg. oil	3	3.84
Lysine sulphate	0.5	0.46
Methionine-DLM	0.27	0.022
L-Theronine	0.01	0.07
Dicalcium phosphate	1	0.89
Calcium carbonate	0.7	0.69
Sodium bicarbonate	0.07	0.12
Sodium chloride	0.24	0.2
Premix^1)^	0.5	0.5
Total	100	100
Nutrients composition (%)		
ME (Kcal/kg)	3,000	3,150
Crude protein	21	19.5
Dig. Lysine	1.18	1.05
Dig. methionine	0.45	0.42
Calcium	0.9	0.84
Available phosphorus	0.45	0.42

ME, metabolizable energy.

1) Vit. A 15,000 IU/kg, B_1_ 2 mg/kg, B_2_ 8 mg/kg, B_6_ 4 mg/kg, B_12_ 1 mg/kg, D_3_ 3,000 IU/kg, E 60 IU/kg, K_3_ 3 mg/kg, pantothenic acid 15 mg/kg, niacin 45 mg/kg, folic acid 1 mg/kg, choline chloride (60%) 500 mg/kg, manganese sulphate 18.5 mg/kg, magnesium sulphate 53 mg/kg, zinc sulphate 2 mg/kg, copper sulphate 45 mg/kg and ferrous sulphate 35 mg/kg.

**Table 2:** Phytochemicals found in *Acacia nilotica* bark extract

Compounds	Availability
Tannins	Present
Terpenoids	Present
Alkaloids	Present
Glycosides	Present
Saponins	Present
Steroids	Absent
Flavonoids	Absent

**Table 3:** Effect of *Acacia nilotica* bark extract on growth performance in broiler

Parameters	Treatments^1)^						SEM	p-value

	NC	AB	PB	ANBE1	ANBE3	ANBE5		
Starter phase								
Feed intake (g)	1,432.81	1,427.08	1,390.68	1,452.43	1,411.45	1,418.16	8.52	0.583
Weight gain (g)	984.65	991.03	970.89	999.46	986.22	991.11	3.89	0.870
FCR	1.46	1.44	1.43	1.46	1.43	1.43	0.00	0.988
Finisher phase								
Feed intake (g)	2,057.63	2,063.14	2054.59	2,017.36	2,042.98	2,038.82	6.79	0.917
Weight gain (g)	1,140.58^b^	1,296.63^a^	1268.21^ab^	1,149.64^ab^	1,214.33^ab^	1,261.43^ab^	26.57	0.017
FCR	1.81^b^	1.59^a^	1.62^ab^	1.77^ab^	1.68^ab^	1.62^ab^	0.04	0.019
Overall								
Feed intake (g)	3,490.44	3,490.22	3,445.27	3,469.79	3,454.43	3,456.97	7.79	0.957
Weight gain (g)	2,125.23^b^	2,287.67^a^	2,239.09^ab^	2,149.10^ab^	2,200.54^ab^	2,252.55^ab^	25.60	0.025
FCR	1.64^b^	1.53^a^	1.54^a^	1.62^b^	1.57^ab^	1.54^a^	0.02	0.001

SEM, standard error of mean; FCR, feed conversion ratio.

1) NC, negative control; AB, antibiotic supplemented group; PB, probiotic supplemented group; ANBE1, ANBE3, and ANBE5 were supplemented with *A. nilotica* bark extract at the levels of 0.1%, 0.3%, and 0.5%, respectively.

a,b Means in a row with different superscript differ significantly (p<0.05).

**Table 4:** Effect of *Acacia nilotica* bark extract on carcass characteristics in broiler

Parameters (%)	Treatments^1)^						SEM	p-value

	NC	AB	PB	ANBE1	ANBE3	ANBE5		
Dressing percentage	68.29	69.21	69.43	68.33	68.40	68.40	0.23	0.080
As a percent of carcass weight								
Breast	22.35	23.50	23.26	21.64	21.53	22.22	0.33	0.177
Thigh	19.58	19.26	19.04	19.56	19.26	19.22	0.09	0.715
Wing	2.16^ab^	2.20^ab^	2.35^ab^	2.18^ab^	2.50^a^	1.89^b^	0.08	0.052
Heart	1.21^c^	1.26^bc^	1.22^bc^	1.32^abc^	1.51^a^	1.45^ab^	0.05	0.003
Liver	3.66	3.86	3.71	3.78	3.86	3.86	0.04	0.885
Gizzard	3.85	3.81	3.96	3.62	3.92	4.17	0.07	0.101

SEM, standard error of mean.

1) NC, negative control; AB, antibiotic supplemented group; PB, probiotic supplemented group; ANBE1, ANBE3, and ANBE5 were supplemented with *A. nilotica* bark extract at the levels of 0.1%, 0.3%, and 0.5%, respectively.

a–c Means in a row with different superscript differ significantly (p<0.05).

**Table 5:** Effect of *Acacia nilotica* leave extract on blood biochemistry in broiler

Parameters (mg/dL)	Treatments^1)^						SEM	p-value

	NC	AB	PB	ANBE1	ANBE3	ANBE5		
Glucose	264.50^a^	229.50^ab^	224.75^b^	240.00^ab^	244.25^ab^	213.75^b^	7.21	0.006
Total protein	2.58	2.62	2.63	2.61	2.48	2.36	0.04	0.295
Albumin	1.36^ab^	1.46^a^	1.33^ab^	1.31^ab^	1.33^ab^	1.20^b^	0.03	0.049
Globulin	1.22	1.16	1.30	1.30	1.15	1.17	0.03	0.874
Triglyceride	61.25	55.25	50.75	48.75	49.75	55.75	1.94	0.357

SEM, standard error of mean.

1) NC, negative control; AB, antibiotic supplemented group; PB, probiotic supplemented group; ANBE1, ANBE3, and ANBE5 were supplemented with *A. nilotica* bark extract at the levels of 0.1%, 0.3%, and 0.5%, respectively.

a,b Means in a row with different superscript differ significantly (p<0.05).

**Table 6:** Effect of *Acacia nilotica* bark extract on intestinal morphology in broiler

Parameters	Treatments^1)^						SEM	p-value

	NC	AB	PB	ANBE1	ANBE3	ANBE5		
Villus height (VH, μm)	1,201.60	1,300.75	1,238.01	1,244.32	1,263.98	1,305.72	16.23	0.091
Crypt depth (CD, μm)	234.83^a^	205.79^b^	219.42^ab^	209.16^ab^	211.03^ab^	204.93^b^	4.64	0.031
VH/CD	5.13^b^	6.34^a^	5.67^ab^	5.95^a^	6.00^a^	6.37^a^	0.19	0.001

SEM, standard error of mean.

1) NC, negative control; AB, antibiotic supplemented group; PB, probiotic supplemented group; ANBE1, ANBE3, and ANBE5 were supplemented with *A. nilotica* bark extract at the levels of 0.1%, 0.3%, and 0.5%, respectively.

a,b Means in a row with different superscript differ significantly (p<0.05).
